# Design of High Impedance Electromagnetic Surfaces for Mutual Coupling Reduction in Patch Antenna Array

**DOI:** 10.3390/ma6010143

**Published:** 2013-01-07

**Authors:** Mohammad Tariqul Islam, Md. Shahidul Alam

**Affiliations:** 1 Institute of Space Science (ANGKASA), Universiti Kebangsaan Malaysia, 43600 Bangi, Selangor, Malaysia; E-Mail: titareq@yahoo.com; 2 Department of Electrical, Electronic and Systems Engineering, Universiti Kebangsaan Malaysia, 43600 Bangi, Selangor, Malaysia

**Keywords:** high impedance surfaces, meandered line, bandgap, array, mutual coupling

## Abstract

A compact planar meander-bridge high impedance electromagnetic structure (MBHIES) was designed and its bandgap characteristics, mutual coupling reduction abilities were studied and compared in detail. Several parametric analyses were performed to obtain optimized design values and the transmission responses were calculated through the suspended microstrip line and waveguide simulation methods. The achieved bandgap is 2.3 GHz (2.55–4.85 GHz) with −61 dB minimum transmission coefficient level at the center frequency of 3.6 GHz. To see the effectiveness, the proposed design was inserted between a microstrip patch antenna array which operates at 3.8 GHz and whose operating bandwidth falls within the MBHIES bandgap. The surface wave suppression phenomenon was analyzed and simulated results are verified by measuring the fabricated prototypes, both are in good agreement. The configuration reduced the mutual coupling by 20.69 dB in simulation and 19.18 dB in measurement, without affecting the radiation characteristics of the array but increasing the gain slightly.

## 1. Introduction

The demand for compact and high performance devices for versatile applications is boosted with the rapid advancement of the wireless technology. Various microwave components such as couplers, dividers, amplifiers, filters, microstrip antennas *etc*. were designed with the microstrip technology, used for high performance applications. Microstrip patch antennas are an attractive and economical solution for designing compact wireless communication devices. Advantages such as light weight, low profile, low cost, and ease of integration with other printed circuits lead to their use in many applications. Several microstrip antennas are arranged in an array to overcome the individual low gain limitation, especially for the applications that requires high gain and high directivity. Mutual coupling between elements of an array adversely affects the radiation characteristics and the overall system performances. The coupling can arise from the excitation of surface waves, space waves and near field overlapping of the array elements. Practically, the coupling mechanisms depend on several factors such as permittivity and thickness of substrate material, ground plane size, type of excited modes *etc*. [1,2]. Numerous techniques were studied and applied to alleviate mutual coupling in an array that includes cavity backing, partial substrate removal, corrugations, split ring resonators (SRR), defected ground structures (DGS), periodic structures like high impedance electromagnetic surfaces (HIES) or electromagnetic bandgap structures (EBG) *etc*. [3,4,5,6,7]. Integration HIES/EBG structures in a microstrip antenna array are quite attractive for their ability to suppress surface waves and reduce the mutual coupling effects. These high impedance surfaces are realized with the periodic arrangement of dielectric or metallic elements. They can efficiently guide and control the propagation of electromagnetic waves at a particular frequency range, which is known as a forbidden frequency band or bandgap [8,9]. These structures reject or block wave propagation within the bandgap while acting as passband beyond the bandgap.

The high impedance structures can be categorized as mushroom-like or uniplanar configuration, where the earlier one includes grounding vias or pins, and later one is totally vialess. Usually, these configurations consist of metallic patches printed on a substrate material and the patches are connected or not to the ground plane through the vias. Mushroom-like HIESs are not always attractive from the electric loss and fabrication perspective while the other one is completely planar, easy and can be fabricated on one layer [8]. Design of a compact planar HIES for lower frequency bands is an optimal challenge. Wideband at lower frequencies is achievable by using larger HIES unit cells but it will increase the volumetric size and hence the fabrication cost. A few planar designs reported previously [10,11] showed bandgap (or stopband) at or higher than 6 GHz. In [12], a planar configuration consists of 920 mils (23.37 mm) unit cell, built on a high permittivity substrate, obtained a bandgap of 1 GHz (2.5 to 3.5 GHz). A multilayer design proposed in [13] for mobile applications, exhibits a bandgap of 0.24 GHz with 16.7 × 3 mm^2^ unit cells.

Many different high impedance structures have been designed based on the mushroom-like configuration described in [1], and applied to reduce mutual coupling of antenna array [3,14,15]. A 3 × 7 mushroom-EBG matrix inserted between a two element microstrip patch antenna array reduced coupling by 10 dB at 5.8 GHz [16]. The fork like EBG proposed in [15] reduced coupling by 6.51 dB than a conventional design. A UC-EBG built on a high permittivity substrate reduced the mutual coupling to −39 dB [17]. Mutual coupling is lowered by 4 dB, at 5.6 GHz with dumbbell-shape structure in [18] and at 5.2 GHz with different shapes of planar designs in [19]. An EBG array of 13.4 × 10.4 mm^2^ C-shaped unit cell inserted between microstrip antennas exhibited 10 dB mutual coupling reductions [20]. Several more other approaches described in the literature reduced the coupling level by 13 dB with UCEBG on the same layer [21], 17 dB with dual-layer [22], and 10 dB with multilayer [23] configurations.

This study proposes a uniplanar compact design of vialess high impedance electromagnetic structure (HIES) with smaller size unit cell. The bandgap characteristics are improved by introducing meander-line connecting bridges instead of straight lines, while the unit cell size is kept fixed. Several parametric studies performed on the proposed design, which revealed wide bandgap at lower frequency bands (below 6 GHz) with a better attenuation level. As the bandgap is positioned at around 3.8 GHz, it can be widely used to improve performances of numerous low frequency applications. The designs are inserted between a two element microstrip antenna array that operates at 3.8 GHz and coupling reduction abilities are verified by comparing the simulated and measured results.

## 2. Design of the HIES

**Figure 1:**
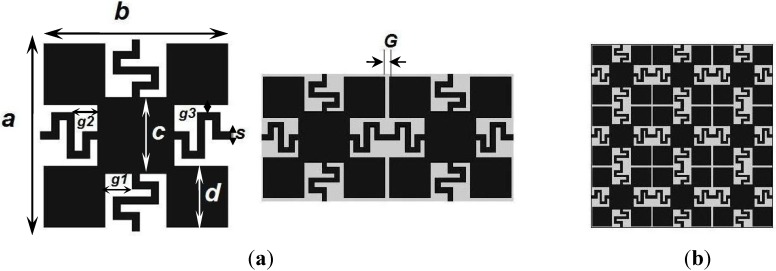
(**a**) Schematic of meander-line bridge high impedance electromagnetic structure (MBHIES) unit cell; (**b**) A 3 × 3 array of proposed MBHIES.

The schematic view of the proposed meander-line bridge high impedance electromagnetic structure (MBHIES) is demonstrated in Figure 1. The optimized shape is obtained after several modifications from a basic rectangular unit cell. The 12.5 × 12.5 mm^2^ unit cell is consists of five square shape patches of two different sizes. A center patch is connected to four other patches on the corner sides. The center patch is larger than the corner patches. Four outwards narrow strips from the center patch establish connecting bridges with the adjacent unit cells. Thus, each unit is connected to four other adjacent cells and provides inductive effects. Initially, the bridges were straight lines, which are modified as meander-line bridge later. The proposed structure is patterned on a 1.6 mm FR4 dielectric material, which has a dielectric constant of 4.5 and a loss tangent of 0.017. As FR4 glass-epoxy is a market available, low cost and commonly used material for a wide range of applications, selection of this material is more convenient.

The high impedance surfaces like EBG exhibits bandgap or stopband characteristics, where the propagation of electromagnetic waves is forbidden or rejected. The rejection band results from the collective balanced inductive-capacitive effect that provided by the artificially perturbed surface. A perturbed surface realized carefully can exhibit band rejection property or higher surface impedance than a conventional flat surface. By properly tuning the design parameters, the bandgap position can be adjusted at the desired frequency band. In this work, the surface modification is done to obtain a stopband below 6 GHz. Due to the complexity of the HIESs, it is usually difficult to characterize them through analytical methods. The proposed structure is designed and optimized by commercially available finite element method (FEM) based full wave electromagnetic simulator HFSS^TM^. The bandgap characteristics of the HIES are studied through different parametric analysis, the structure is modified and the performances are investigated.

**Figure 2:**
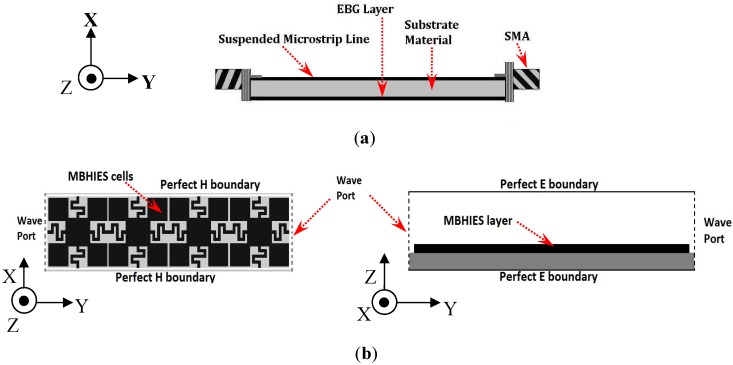
Bandgap prediction by (**a**) Suspended transmission line method; (**b**) Waveguide transmission method.

The bandwidth of the stopband (or bandgap) of the structure is computed by the suspended microstrip transmission line method (SML) [15] and waveguide simulation (WGM) (or directive transmission) method [24], with an array of a finite number of unit cells. The simulation models are as shown in Figure 2. In the first method, a 2.8 mm wide suspended microstrip line with 50 Ω characteristic impedance is printed on the opposite layer of the substrate as shown in Figure 2a. In the presence of the HIES layer, the transmission responses (*S_21_*) between the two ports are analyzed to obtain the bandgap properties. In the other method, the structure is simulated within a two-port waveguide and the transmission responses are computed along the main direction of periodicity (*Γ-Χ* direction). The TEM waveguide is realized with a pair of perfect E boundaries (electric walls) and perfect H boundaries (magnetic walls). Normal incident waves from each port are launched into the free space towards the waveguide. Four HIES unit cells are inserted within the waveguide along Y-axis.

**Figure 3:**
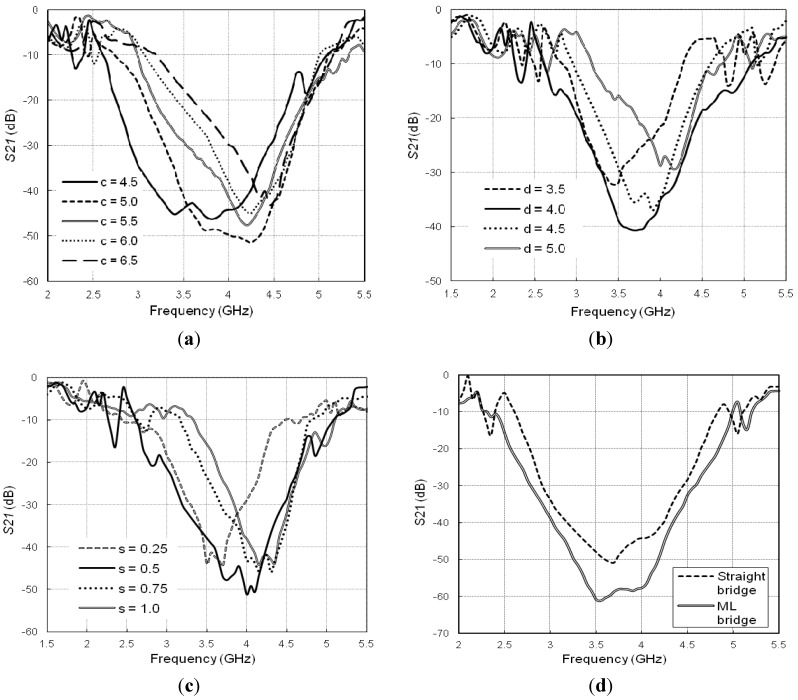
Transmission responses with variation of the (**a**) Centre patch size; **(b**) Corner patch size; (**c**) Bridge width; (**d**) straight bridge vs. meander-line (ML) bridge.

Several parametric studies have been performed on the design parameters such as bridge width (*s*), central (*c*) and corner patch (*d*) sizes. One parameter variation is considered at a time while the others remain fixed. Figure 3a shows the variation of the transmission characteristics for different center patch sizes (*c*). The patch size varies from 4.5 × 4.5 mm^2^ to 6.5 × 6.5 mm^2^. With increasing patch size, the center frequency shifts to higher band and the *S_21_* level degrades gradually. The bandwidth of the stopband also narrows down with the patch larger than 5 × 5 mm^2^. A variation of the center patch size changes the connecting bridge length, hence the surface impedance varies which in turn affects the bandgap of the HIES structure. The *S_21 _* is varied within −39 to −50 dB, the lowest value obtained is −60 dB at 3.9 GHz and the width of bandgap is 1.98 GHz, with a 4.5 × 4.5 mm^2^ center patches.

The effect of the different corner patch sizes on the HIES bandgap is also examined by varying the design parameter *d* (Figure 3b). As the gap between the connecting bridges and the corner patches directly vary with corner patch area, the gap capacitances become a function of *d.* For different *d* values*,* the center frequency and the minimum *S_21_* level varied within the range of 3.5 to 4.25 GHz, and −38 to −51 dB, respectively. The HIES unit with 4 × 4 mm^2^ corner patches results a wider bandgap of 1.45 GHz (3–4.45 GHz) with a minimum transmission level of -41 dB. Another important parameter is the bridge width (*s)* which shows significant control over the bandgap characteristics (Figure 3c). A narrow bridge of 0.25 mm width, exhibits a bandgap of 1.1 GHz at 3.6 GHz with a minimum *S_21_* of −44 dB. As gradually the width increased to 1 mm, the center frequency also changes position to 4.3 GHz, though the bandwidth is not much affected. However, compared to the other values, *s* = 0.5 mm gives a deeper and wider bandgap at 3.8 GHz. Because the connecting bridges play important role in determining the bandgap position as well as the bandwidths, the straight microstrip bridges are modified to meander line shape. The 0.5 mm wide meander lines increase the effective bridge lengths and add more inductances that contribute to raise the total surface impedance. It is worth noting that the bandgap and the rejection level (dB) significantly depends on the combination of the all important design parameters. The connecting bridges provide the inductances while the gaps within a cell and the gap between adjacent cells provides the capacitances, thus the configuration exhibits the high impedance nature. Thus, a proper combination of the design parameters is significant to obtain wide bandgap at desired frequency ranges. A trade-off is considered among the parameters to achieve best possible results and the selected parameters are given below:

a = 12.5; b = 12; c = 4.8; d = 4.1; e = 3.85; g_1_ = 1.65; g_2_ = 1.5; g_3_ = 0.55; s = 0.5; G = 0.5 (1)

With these parameters, the transmission responses of the HIES with straight bridge and meander line bridge are compared in Figure 3d. The improvement of bandwidth and transmission level is easily understandable. The stopband is extended from 2.55 to 4.85 GHz (2.3 GHz) and centered at 3.6 GHz. The minimum level of *S_21_* is lowered by 11 dB, reaches to −61 dB. With respect to the center frequency, the fractional bandwidth of the MBHIES bandgap is 62.16%, which is 24.32% (450 MHz) higher than the straight bridge design (1.85 GHz, 49.66%).

**Figure 4:**
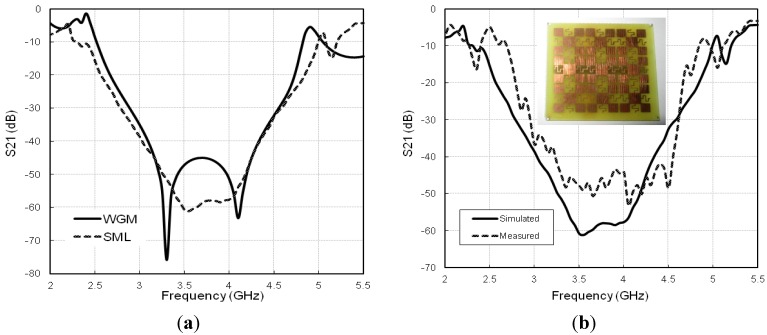
(**a**) Transmission responses of the MBHIES from two different methods; (**b**) Simulated and measured transmission responses, *S_21_*.

Figure 4a compares the bandgap characteristics of the MBHIES investigated through two different methods. Both of the methods show the existence of a wide bandgap with periodicity along the main propagation direction *Γ-Χ.* To validate the simulated results, a 3 × 3 array of MBHIES is manufactured with a printed suspended microstrip line on the opposite side of the substrate. The transmission responses between the two ports are measured by Agilent’s 8362C PNA vector network analyzer and the measured spectra is compared with simulated one in Figure 4b. Despite of slight mismatches due to fabrication and soldering tolerances, measured result is in good agreement with its simulated counterpart.

## 3. Mutual Coupling Reduction of Microstrip Antenna Array

**Figure 5:**
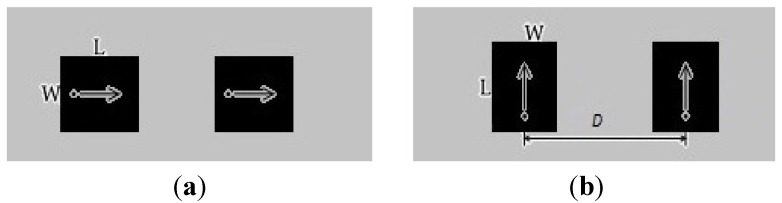
Probe fed microstrip antenna array (**a**) E-plane coupling; (**b**) H-plane coupling.

Microstrip patch antennas are often arranged in array for high gain, beam forming or diversity purposes [25]. However, the pronouncement of surface waves and overlapping of near fields of the array elements lead to strong mutual coupling, which severely degrades the antenna performances. Usually, the E-plane coupled antennas suffer from a stronger coupling effect than the H-plane coupled antennas, because the surface waves strongly propagate along the E–plane direction [10]. Increasing substrate thickness and permittivity also increases the mutual coupling. Figure 5 illustrates the coupling direction of a probe-fed microstrip patch antenna array. Among the several other methods for example cavity backing, substrate removal *etc*., the HIES/EBG structures exhibit better results to reduce the mutual coupling between array elements.

**Figure 6:**
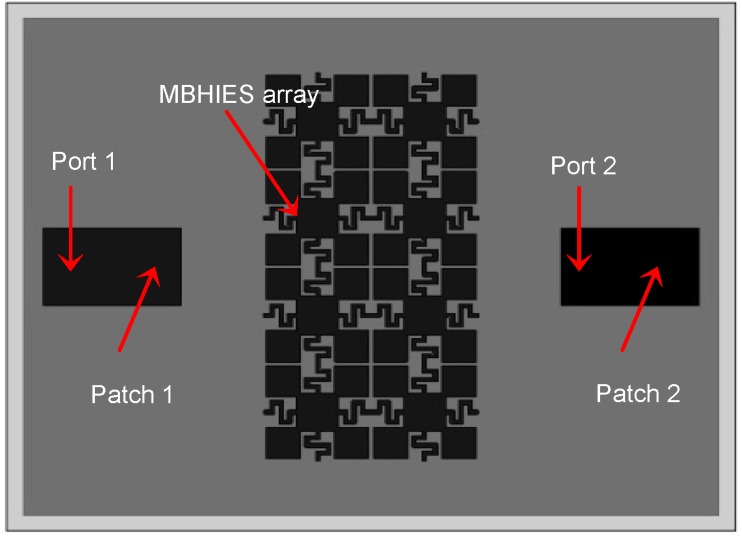
E-plane coupled microstrip antenna array with MBHIES.

The effectiveness of the proposed MBHIES design is investigated by applying an E-plane coupled microstrip patch antenna array. The antennas are designed to operate at 3.8 GHz, which falls inside the bandgap of the high impedance structures. Each antenna element has a dimension of 22 × 27 mm^2^ and the separation between the antennas is *D = 0.45λ* at 3.8 GHz, where *λ* is the free space wavelength. In between the radiating patches, eight MBHIES unit cells are placed in two columns as shown in Figure 6. The coupling reduction effects of the MBHIES are investigated and compared with the reference antenna array consequently. During the comparison, the antenna size, substrate properties and antenna distance in all the structures are kept the same as in the normal case.

**Figure 7:**
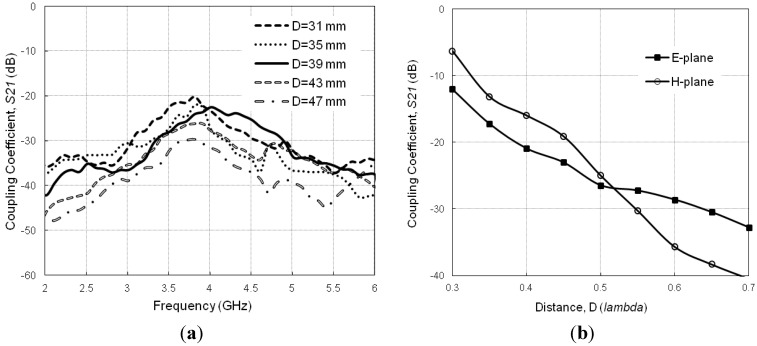
Coupling coefficient, *S_21_* as a function of patch separation, *D* (**a**) *S_21_* vs. frequency; (**b**) minimum* S_21_* vs*. D* for both E and H-plane directions.

Prior to applying MBHIES to the antenna array, the mutual coupling effects are examined by varying the patch separation (*D*). Figure 7a plots the mutual coupling for various patch separations and Figure 7b plots the minimum coupling (in both E- and H-plane directions) for different distances at the resonant frequency of 3.8 GHz. It is clear from the figures that the coupling coefficient level degrades with increasing distance between array elements in both directions. Although the antennas become less coupled with increasing distance, it will increase antenna volumetric size. Therefore, application of high impedance periodic structures is a viable solution for this case.

**Figure 8:**
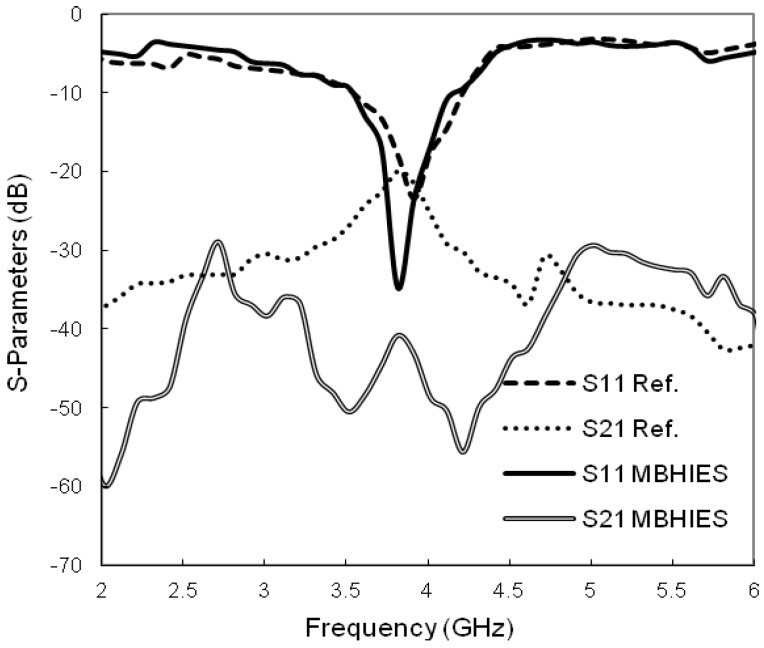
Simulated *S-*parameters of the microstrip antenna array.

Figure 8 shows the effect of the MBHIES on the mutual coupling of the patch antenna array. Without the structure, the antennas show a strong coupling (*S_21_*) of −20.10 dB at 3.8 GHz. When the proposed structures are employed in the array the coupling level changes noticeably. The mutual coupling level is reduced to −40.79 dB, without changing the resonance nature of the array. It can be noted that the bandwidth of the periodic structure is much wider than the operational bandwidth of the antenna, thus it sufficiently suppressed the surface waves inside the bandgap. Therefore, the coupling effect is reduced by 20.69 dB. The −10 dB impedance bandwidths are not much affected except slight shifting in the resonance; moreover, deeper reflection coefficient (*S_11_*) levels are achieved.

**Figure 9:**
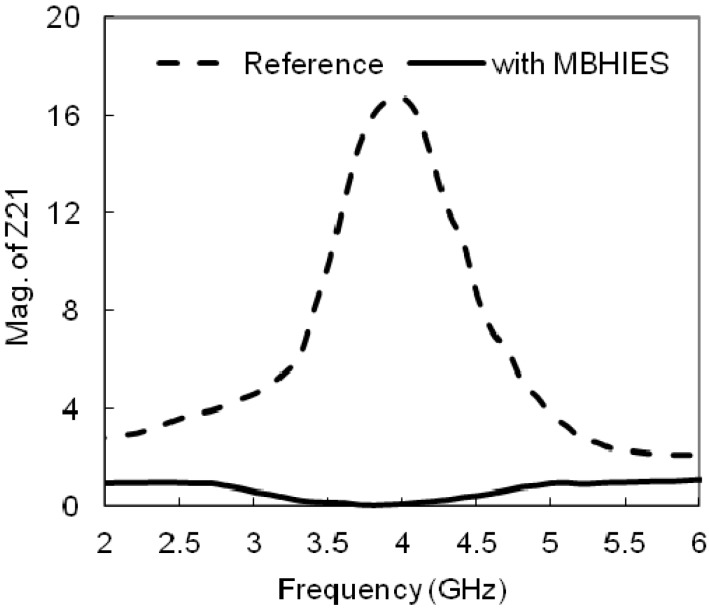
Simulated magnitude of *Z_21_* for the proposed HIES design.

**Figure 10:**
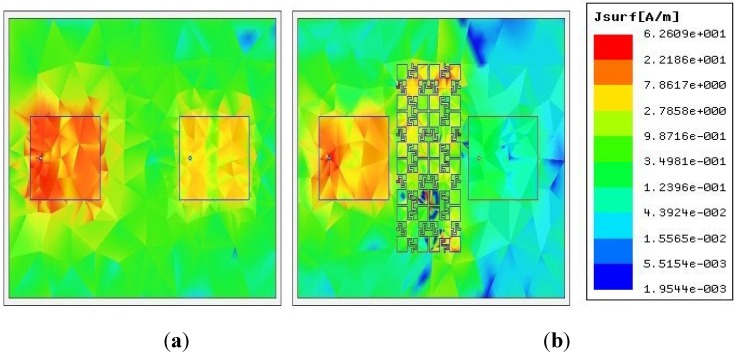
Snapshots of the surface current distribution (**a**) reference array; (**b**) with MBHIES.

The mutual impedance (*Z_21_*) between two the patches is shown in Figure 9. It is nearly zero in the HIES case, while showing magnitude higher than 16 in the normal case, which also demonstrate the coupling reduction between two elements. This comparison demonstrates the capability of the high impedance periodic structures to reduce the mutual coupling. The pronouncement of the surface waves is restricted by the filtering performance of the inserted MBHIES cells; hence the microstrip patches are less coupled than the normal case. The mutual coupling phenomenon can further be clarified by observing the surface current distribution. The surface current distribution of the patch antenna array is plotted in Figure 10, where one patch is excited and the other one is terminated with 50-Ω impedance. In normal case, high concentration of surface current is seen, while it is well suppressed by the MBHIES cells. Therefore, the antennas are being less coupled than normal case.

**Figure 11:**
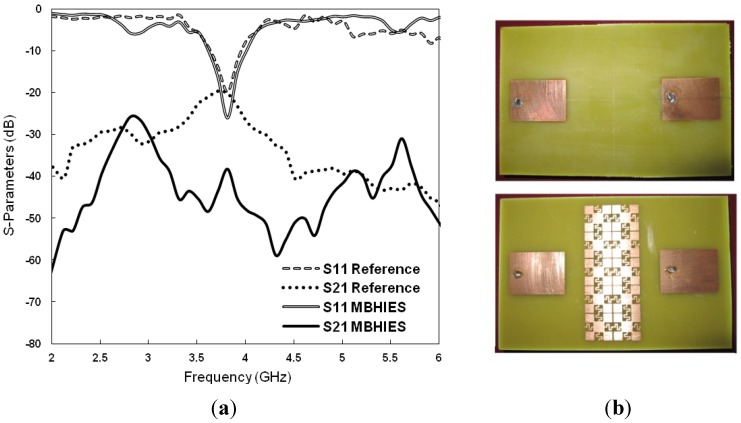
(**a**) Measured *S-*parameters of the microstrip antenna array; (**b**) fabricated prototypes.

Finally, the array designs are fabricated and measured by using Agilent-E8362C PNA vector network analyzer. Figure 11 shows photographs of the manufactured prototypes and the measured *S-*parameters of them. The resonance frequency shifted slightly to the lower side, though the *S_11_* levels are still deeper. The mutual coupling level is nearly −19.51 dB between the normal array design and −38.69 dB with the MBHIES configuration, at 3.8 GHz. The measured results show very good agreement with the simulated results, except slight discrepancies, which can be attributed to the fabrication and soldering imperfection.

**Figure 12:**
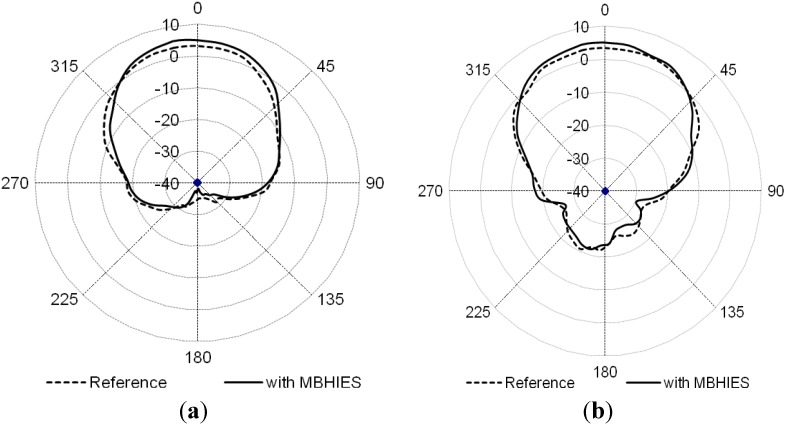
Gain patterns of the array with and without MBHIES at 3.8 GHz: (**a**) E-plane; (**b**) H-plane.

The gain patterns of the microstrip antenna array are depicted in Figure 12. In the both the E- and H-plane, radiation characteristics with and without MBHIES are compared at 3.8 GHz. Inclusion of the high impedance surfaces does not affect the radiation characteristics, though it enhanced the antenna gain slightly and improved the isolation between the radiating patches.

## 4. Conclusions

This work presents a compact design of vialess planar MBHIES with wide bandgap and deeper rejection levels. Insertion of meander line bridges instead of the straight lines enhanced the HIES performances with respect to the bandgap and rejection value. The bandgap properties were computed through suspended microstrip line and waveguide transmission (directive transmission) methods for better understanding. The resulting bandgap is 2.3 GHz (2.55–4.85 GHz) at −20 dB transmission coefficient value, which is 62.16% wide with respect to the center frequency (3.7 GHz). In the low frequency bands (< 6 GHz), the minimum level of transmission coefficient is −61 dB at 3.6 GHz. The proposed structure was applied to a microstrip patch antenna array, whose operating frequency band (3.6–4.1 GHz) is covered by the bandgap (2.55–4.85 GHz) of the high impedance structure. The coupling reduction mechanism was thoroughly analyzed and discussed, with various results. Significant reduction of mutual coupling of antennas is achieved (20 dB) by the MBHIES, while the radiation characteristics remain unaffected. The simulated results are validated by measuring manufactured prototypes and comparing them. Counting on the performances, the proposed configurations could be a good choice for remarkable number applications of microstrip antenna array that operates in the lower bands.
